# Optimized protocol to detect protein UFMylation in cells and in vitro via immunoblotting

**DOI:** 10.1016/j.xpro.2021.101074

**Published:** 2022-01-06

**Authors:** Junzhi Zhou, Qian Liang, Maogong Dong, Xiaohe Ma, Yaqi Jin, Di Guan, Jiang Liu, Miao Wang, Yu-sheng Cong

**Affiliations:** 1 Key Laboratory of Aging and Cancer Biology of Zhejiang Province, School of Basic Medical Sciences, Hangzhou Normal University, Hangzhou, China

**Keywords:** Cell Biology, Molecular Biology, Protein Biochemistry

## Abstract

Ubiquitin-fold modifier 1 (UFM1) system is a recently identified ubiquitin-like modification with essential biological functions. Similar to ubiquitination, the covalent conjugation of UFM1 (UFMylation) to target proteins involves a three-step enzymatic cascade catalyzed sequentially by UFM1-activating enzyme 5 (UBA5, E1), UFM1-conjugating enzyme 1 (UFC1, E2), and UFM1-specific ligase 1 (UFL1, E3). Here, we provide an optimized protocol adapted to previously reported methods for detecting the UFMylation of target protein in human cells and *in vitro* assays*,* respectively, with high reliability and reproducibility.

For complete details on the use and execution of this protocol, please refer to Liu et al. (2020).

## Highlights

• UFMylation modification involves a three-step enzymatic cascade
• Protocol established for analysis of the UFMylated protein in cells and *in vitro*
• Efficient protocol for identification of potential UFMylation target proteins

## Graphical abstract

**Figure:**
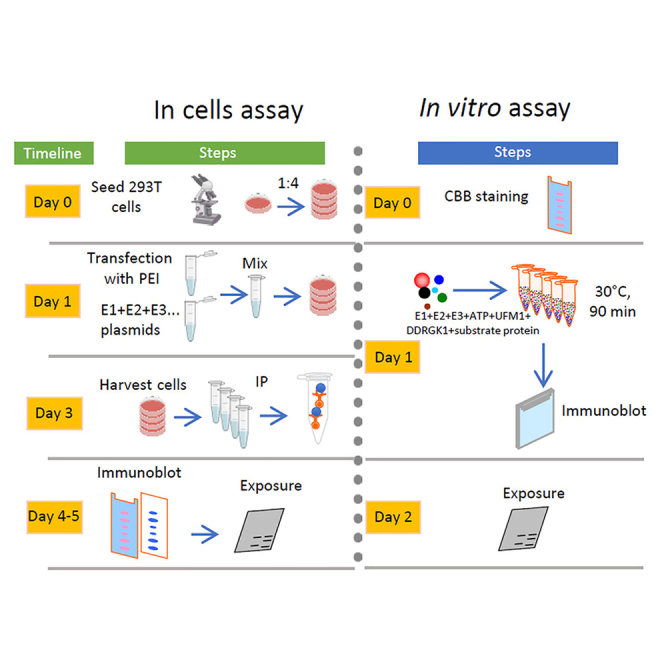

## Before you begin

Preparing high quality plasmid DNAs and HEK293T cells for UFMylation assay in cells, and preparing recombinant proteins of each UFMylation components for UFMylation assay *in vitro.*

This protocol describes a detailed procedure for UFMylation analysis in cells and *in vitro* assay. UFMylation is a recently identified ubiquitin-like modification, which covalently conjugates the ubiquitin-like protein UFM1 to target proteins through a three-step enzymatic cascade catalyzed sequentially by UFM1-activating enzyme 5 (UBA5, E1), UFM1-conjugating enzyme 1 (UFC1, E2), UFM1-specific ligase 1 (UFL1, E3) and cofactor DDRGK1 (Komatsu et al., 2004; Gerakis et al., 2019). For UFMylation assay in human cells, we transiently transfect the expression plasmids of UFMylation component (E1, E2, E3, DDRGK1 and UFM1) and the Flag-tagged substrate protein (FLAG-p53) into HEK293T cells, followed by immunoprecipitation with Flag beads and immunoblotting to detect the UFMylated protein with the UFM1 antibody. For *in vitro* UFMylation assay, we reconstitute UFMylation reaction system with the purified UFMylation components *in vitro*, followed by immunoblotting to detect the UFMylated protein.

### Endotoxin-free plasmids

**Timing: 2 days**

Preparation of plasmid DNAs for UFMylation assay in cells. HA-tagged UFMylation component expression plasmids (pSG5-HA-UFM1, pSG5-HA-UFM1-ΔC2, pSG5-HA-UFM1-ΔC3, pSG5-HA-UBA5, pSG5-HA-UFC1, pSG5-HA-UFL1, pSG5-HA-DDRGK1, and pSG5-HA-vector) and FLAG-tagged substrate expression plasmid (pCDNA3.0-FLAG-His-p53 and pCDNA3.0-FLAG-His-vector) were prepared from 200 mL *E. coli* which had been cultured for 10–12 h by using Endotoxin-free plasmids kit (TIANGEN). The concentrations of plasmids were measured by NanoDrop One spectrophotometer (Thermo Fisher). Plasmids can be stored at −20°C in small aliquots.

### HEK293T cell seeding and cell passage

**Timing: 2–3 days**

1. Put 1–2 vials from liquid nitrogen in a 37°C water bath with shaking until completely defrosted.
2. Transfer thawed cells into a 15 mL sterile conical tube prefilled with 4 mL complete DMEM (DMEM supplemented with 10% fetal bovine serum, 1% L-glutamine, 100 U/mL penicillin and 100 μg/mL streptomycin), and centrifuge at 150×*g* for 3 min, then carefully aspirate supernatant by a vacuum pump.
3. Re-suspended cells in a total 10 mL fresh complete DMEM in a 10 cm dish and culture in a 37°C incubator with a humidified atmosphere of 5% CO2.
4. HEK293T cells are typically passaged when 80%–90% confluent to a ratio of 1:4.

### Cell preparation

**Timing: 4–5 days**

5. Aspirate the culture medium and add 5–10 mL of sterile PBS to rinse the cells.
6. Aspirate the PBS and add 1 mL Trypsin EDTA solution A (0.25%) to detach cells in the cell incubator at 37°C.
7. Add 5 mL of complete DMEM and resuspend the cells. Collect the cell suspension in a 15 mL conical tube.
8. Centrifuge at 150×*g* for 3 min, then carefully aspirate supernatant by a vacuum pump.
9. Resuspend cells in 40 mL complete DMEM and divide evenly into four 10 cm diameter plates.

***Note:*** One 10 cm-dish with 90%–100% confluent HEK293T cells provides sufficient cells for one sample UFMylation detection. Therefore, the amount of 10 cm dishes should be calculated according to the experiment plan. Before transfection, expand sufficient amounts of HEK293T cells.

### Preparation of recombinant protein for UFMylation assay in vitro

**Timing: 5 days**

***Note:*** Most recombinant proteins for UFMylation assay *in vitro* are commercially available, we have prepared in house recombinant UFL1 and DDRGK1 from *E.coli*, other components (UBA5, UFC1, UFM1, the substrate p53) were purchased from R&D Systems.

10. Culture BL21 (DE3) *E. coli* cells harboring the pGEX-6P-1-DDRGK1 or pGEX-6P-1-UFL1 plasmid in 1 L LB at 37°C for 3–5 h with rotation at 220 rpm to a cell density (OD_600_) of 0.6.
11. Add 5 mL IPTG (100 mM) into the *E. coli* culture medium to final concentration of 0.5 mM with shaking at 19°C for 16–18 h.
12. Transfer *E. coli* culture to 200 mL centrifuge tubes and spin at 8,000×g for 5 min at 4°C, then discard the supernatants carefully.
13. Resuspend the cell pellet in 50 mL pre-cold PBS with 1× protease inhibitor cocktail (Roche) and 1 mM PMSF.
14. Extract protein by breaking bacterial cells using cell disrupter at high pressure for 2–3 cycles.
15. Centrifuge the crude lysate at 14,000×*g* for 30 min at 4°C.
16. Equilibrate the GST Sepharose gravity column (1 mL) with 5 mL (5 column volume, 5CV) pre-cold 1× PBS with 0.5% TritonX-100 (PBS-X).
17. Load an approximate 50 mL of supernatant (collected from step f) onto the GST Sepharose gravity column.
18. Wash the column with 15 CV PBS-X.
19. Drain the column by gravity and incubate the column with 1 mL PBS-X containing PreScission Protease at 4°C for 16 h.
20. Collect the column flow-through, add glycerol to 20% final concentration, aliquot and store at −80°C.

***Note:*** All the recombinant proteins required for reconstitution of UFMylation *in vitro* assay are currently commercially available (see the key resources table).

### Preparation of polyethyleneimine (PEI) solution

**Timing: 4 h**

PEI, a synthetic cationic polymer, can complex with negatively charged macromolecules such as nucleic acids to form small particles capable of gene transfection into various cell lines.

21. Take about 450 mL distilled and sterilized water into a 500 mL glass beaker.
22. Dissolve 500 mg of PEI in the water while stirring.
23. Add approximately 1 mL concentrated HCl (12 M) with a plastic dropper into the solution to bring pH <2.0.
24. Stir for 2–3 h until the PEI is fully dissolved.
25. Add 1–2 mL NaOH (3 M) stock buffer with a plastic dropper into the solution to bring pH 7.0.
26. Add the solution into a 500 mL glass cylinder. Adjust the final volume to 500 mL with above water.
27. Sterile filter the solution through 0.22 μm membrane using a sterile 20 mL syringe.
28. Aliquot PEI in 1 mL/1.5 mL EP tubes and store at −20°C.

**CRITICAL:** The concentrated hydrochloric acid is an irritating and toxic fume that causes severe respiratory irritation. Similarly, NaOH solution may cause skin and eyes irritation. Personal protective equipment is required.

### Polyethyleneimine (PEI) transfection solution

**Table:** 

Reagent	Final concentration	Amount
Polyethyleneimine	1 mg/mL	100 mg
HCl (12 M)	N/A	Require
NaOH (3 M)	N/A	Require
ddH_2_O	N/A	Up to 100 mL
**Total**	N/A	**100 mL**

***Note:*** The PEI solution can be stored at −20°C for up to one year. We recommend using the PEI solution for HEK293T cell transfection. The protocol for the PEI transfection solution was from Cold Spring Harbor Protocol (http://cshprotocols.cshlp.org/content/2008/3/pdb.rec11323.full).

## Key resources table

**Table:** 

REAGENT or RESOURCE	SOURCE	IDENTIFIER
**Antibodies**		

GAPDH	HuaAn Biotechnology	M1310-2
FLAG	Sigma-Aldrich	F7425
HA	Cell Signaling Technology	3724S
UFM1	Abcam	Ab109305
Anti-Flag M2 Affinity Gel	Sigma-Aldrich	A2220
Goat polyclonal anti-mouse-HRP	Sigma-Aldrich	A9169
Goat polyclonal anti-rabbit-HRP	Sigma-Aldrich	A9044
Anti-p53	Santa Cruz	sc-126

**Bacterial and virus strains**		

DH5α	TIANGEN	CB101
BL21 (DE3)	TIANGEN	CB105

**Chemicals, peptides, and recombinant proteins**		

Dulbecco’s Modified Eagle’s Medium(DMEM)	Biological Industries	06-1055-57-1ACS
Fetal Bovine Serum (FBS)	Biological Industries	04-001-1ACS
Trypsin EDTA solution A (0.25%)	Biological Industries	03-050-1A
Opti-MEM™ I Reduced Serum Medium(Opti-MEM)	Gibco	31985070
Penicillin-Streptomycin	Biological Industries	03-031-1B
Polyethyleneimine (PEI)	Polysciences	23966
N-Ethylmaleimide (NEM)	Sigma-Aldrich	E3876
Imidazole	Biosharp	1-1025
NP40 Alternative	Sigma-Aldrich	492016
cOmplete Protease Inhibitor Cocktail Tablets	Roche	11697498001
Sodium Chloride	Sigma-Aldrich	7647-14-5
PMSF	Roche	329-98-6
SDS	Sigma-Aldrich	151-21-3
MgCl_2_	Sigma-Aldrich	7786-30-3
Recombinant Human His6-UFM1 Activating Enzyme (UBA5)	R&D Systems	E-319-025
Recombinant Human His6-UFC1 Protein (UFC1)	R&D Systems	E2-675-100
Recombinant Human His6-UFM1 Protein (UFM1)	R&D Systems	UL-500-250
p53 Protein	R&D Systems	SP-454-020
Recombinant Human UFL1	Abnova	H00023376-P01
Human DDRGK1 protein (Recombinant Myc-DDK (FLAG)) (Full Length)	LSBio	LS-G72211-20
IPTG	Sigma-Aldrich	16758

**Critical commercial assays**		

SuperSignalTM West Femto Maximum Sensitivity Substrate	Thermo Fisher Scientific	34095
ATP (10 mM)	Cell Signaling Technology	9804S
PreScission protease	Beyotime	P2303

**Experimental models: Cell lines**		

HEK293T cells	ATCC	CRL-11268

**Recombinant DNA**		

pSG5-HA	Liu et al. (2020)	N/A
pSG5-HA-UFM1	Liu et al. (2020)	N/A
pSG5-HA-UFM1-ΔC2	Liu et al. (2020)	N/A
pSG5-HA-UFM1-ΔC3	Liu et al. (2020)	N/A
pSG5-HA-UBA5	Liu et al. (2020)	N/A
pSG5-HA-UFC1	Liu et al. (2020)	N/A
pSG5-HA-UFL1	Liu et al. (2020)	N/A
pSG5-HA-DDRGK1	Liu et al. (2020)	N/A
pCDNA3.0-FLAG-His	Liu et al. (2020)	N/A
pCDNA3.0-FLAG-His-p53	Liu et al. (2020)	N/A
pGEX-6P-1	Liu et al. (2020)	N/A
pGEX-6P-1-UFL1	Liu et al. (2020)	N/A
pGEX-6P-1-DDRGK1	Liu et al. (2020)	N/A

**Software and algorithms**		

Image Lab	Bio-Rad Laboratories	https://www.bio-ad.com

**Other**		

Heraeus Multifuge X3R	Thermo Fisher Scientific	41270886
Centrifuge	Eppendorf	5424R
Centrifuge	Eppendorf	5424
ChemiDoc^TM^ MP Imaging System	Bio-Rad Laboratories	734BR4093
Ultrasonic Cell Disruptor	Tengxiao	TX650

### Key assay buffers and solutions

**Table:** Lysis buffer

Reagent	Final concentration	Amount
pH8.0 Tris-HCl (1 M stock)	150 mM	15 mL
SDS	5% (w/v)	5 g
Glycerol (100% stock)	30%	30 mL
ddH_2_O	N/A	55 mL
**Total**	N/A	**100 mL**

***Note:*** Store at 20°C–25°C for up to one year.

**CRITICAL:** SDS may cause skin and respiratory irritation and should be weighed in the fume hood. Personal protective equipment including rubber gloves and face mask are required.

**Table:** Buffer A

Reagent	Final concentration	Amount
pH8.0 Tris-HCl (1 M stock)	50 mM	25 mL
NaCl (5 M stock)	150 mM	15 mL
Imidazole (2 M)	10 mM	2.5 mL
NP-40	0.5%	2.5 mL
1× protease inhibitor	N/A	N/A
NEM (1 M stock)	2 mM	1 mL
ddH_2_O	N/A	454 mL
**Total**	N/A	**500 mL**

***Note:*** The buffer can be stored at 4°C for up to one year. To minimize degradation of extracted proteins, make sure that protease inhibitor and NEM are freshly added into the buffer A.

## Step-by-step method details

### Assay for UFMylation in cells

**Timing: 48 h**

We have adopted previously reported protocols (Komatsu et al., 2004; Tatsumi et al., 2010; Ishimura et al., 2017) with optimization. In addition, we have included an active (UFM1ΔC2) and defective (UFM1ΔC3) UFM1 for UFMylation detection and data interpretation.

The following steps describe the detailed procedure to efficiently transfect HEK293T with HA-tagged UFMylation component expression plasmids and FLAG-tagged substrate expression plasmid DNAs using PEI transfection solution.

1. Plasmid transfection
  a. HEK293T cells were seeded the night before and transfected with plasmids DNA around 8–10 h later.
  b. In the next morning, transfect cells reached 50%–60% confluency with plasmids using PEI transfection solution. Pre-warm complete DMEM and Opti-MEM in a water bath at 37°C and thaw a fresh aliquot of PEI, HA-tagged UFMylation expression plasmids, FLAG-tagged p53 expression plasmid, and empty vectors in cell culture hood.
  c. Add pre-warmed 500 μL Opti-MEM in the tube A.
  d. Add 54 μL PEI transfection solution in the tube A and mix by gently tipping the tube.
  e. Take HA-tagged expression plasmids of UBA5 (2 μg), UFC1 (2 μg), UFL1 (2 μg), DRGK1 (2 μg), UFM1 (6 μg), and FLAG-tagged p53 expression plasmid (2–6 μg) in a separate tube B.
  f. Add pre-warmed 500 μL Opti-MEM to tube B and mix well by tipping the tube.
  g. Add diluted DNA of tube B to diluted PEI of tube A and mix well.

**Table:** 

	Tube A		Tube B		
	Reagents	Amount	Reagents	Amount	Ratios of DNAs
Step 1			pSG5-HA-UBA5	2 μg	1
			pSG5-HA-UFC1	2 μg	1
	PEI solution	54 μL	pSG5-HA-UFL1	2 μg	1
			pSG5-HA-DDRGK1	2 μg	1
			pSG5-HA-UFM1	6 μg	3
			pCDNA-FLAG-His-p53	2–6 μg	1–3
	Opti-MEM	500 μL	Opti-MEM	500 μL	
Step 2	Mix diluted DNA of tube B to diluted PEI of tube A

    ***Note:*** The optimized amounts, ratios of reagents, and plasmids were listed. For different target proteins, adjust the amount of plasmid DNA that does not exceed 20 μg in total. PEI transfection solution is used for high efficiency transfection in HEK293T cells. However, alternative transfection approaches including calcium phosphate transfection or lentivirus mediated gene delivery may be considered.
  h. Incubate for 15 min at 20°C–25°C. Meanwhile, replace the medium with 8 mL fresh complete DMEM medium.
  i. Add the DNA-PEI complex dropwise to cells and gently rotate the dish to mix the transfection mix and medium. Return plates to the incubator.
  j. At 6–8 h after transfection, gently aspirate the culture medium and add 15 mL fresh complete DMEM to each 10 cm dish.
  k. Harvest cells 48 h after transfection.
    ***Note:*** Pre-warm the complete DMEM and Opti-MEM with a 37°C water bath. Pre-thaw PEI transfection solution and plasmids in cell culture hood and avoid repeated freeze-thaw cycles.

2. Cell collection, lysis and immunoprecipitation
  **Timing: 14–16 h**
  a. Aspirate culture medium and gently rinse cells once with 6 mL PBS. Aspirate the PBS and thoroughly remove the residual liquid.
  b. Add 400 μL lysis buffer dropwise to cells, collect cells using a plastic scraper and transfer them into a 1.5 mL EP tube.
  c. Lyse cells by boiling for 7–10 min at 100°C, then centrifuge at 14,000×*g* for 1 min at 20°C–25°C to collect the supernatant.
  d. Add 900 μL Buffer A into the supernatant and mix well by vortex for 10 s.
  e. Centrifuge at 14,000×*g* for 20 min at 4°C.
  f. Take out 80 μL supernatant (collected from step e) of each sample as input, and transfer the rest to a 15 mL conical centrifugal tube which is pre-filled with 8 mL ice-cold buffer A.
  g. Add pre-washed beads evenly into the above 15 mL tube and vortex with a rotator at 4°C for 10–12 h.
  h. Add 1× SDS loading dye to the input and boil for 10 min at 100°C, then store at −20°C freezer for use.
  i. In the next morning, Spin down the overnight incubated beads at 800×*g* at 4°C for 5 min and aspirate the supernatant.
  j. Add 10 mL pre-cold buffer A into the 15 mL tube and mix by tapping, then centrifuge at 800×*g* at 4°C for 5 min. Then, discard the supernatant carefully and repeat the wash step 5 times.
  k. Add 160 μL of 2× SDS loading buffer into beads for each sample and mix well, boiling for 10 min at 100°C.
  l. Centrifuge at 14,000×*g* for 2 min at 20°C–25°C and transfer approximate 160 μL of supernatant into a new 1.5 mL EP tube as IP sample, then store at −20°C freezer.
  m. Preparation of Anti-Flag M2 Affinity Gel during the period of the centrifuge (step e) as following:
    i. Vortex beads stock solution thoroughly for 30 s to make a homogeneous slurry.
    ii. Transfer the beads (30 μL beads/sample) into a 1.5 mL EP tube using a pre-trimmed 200 μL pipette tip.
    iii. Centrifuge at 800×g for 3 min at 4°C, carefully discards the supernatant.
    iv. Add 1 mL buffer A to the tube and mix by tapping and centrifuge at 800×g for 3 min at 4°C, carefully aspirate the supernatant.
    v. Resuspend beads with 1 mL buffer A and put it on ice.
      ***Note:*** To reduce the loss of beads during the washing steps, we recommend to aspirate most of the supernatant with the vacuum pump, and keep 100–200 μL of liquid at the bottom of the 15 mL tube. Aspirate the residual liquid by 200 μL pipette along the wall of the tube.

3. Detection of UFMylated proteins by immunoblotting
  **Timing: 24 h**
  a. Input (5 μL) and IP (20 μL) samples were separated on 10% SDS-PAGE gel and then transferred to 0.22 μM PVDF membrane.
    ***Note:*** PVDF membranes should be immersed in a methanol bath for 1 min with a shaker for activation of PVDF membrane before transfer of protein samples from SDS-PAGE.
  b. The PVDF membranes were blocked by 5 % (w/v) nonfat dry milk for 1 h at 20°C–25°C and incubated with primary antibodies at 4°C on a shaker for 10–12 h.
  c. Collect the primary antibodies.
  d. Rinse PVDF membranes with 20 mL TBS with 0.1% tween-20 (TBST) for 5 min on a shaker 3 times.
  e. Incubate membranes with secondary antibodies for 1 h on a shaker at 20°C–25°C .
  f. Rinse PVDF membranes as described above.
  g. Prepare fresh ECL solution and incubate PVDF with ECL solution for 30–60 s at 20°C–25°C .
  h. Adjust the chemiluminescence signals and record images.
    ***Note:*** For the very weak signals, we recommend that a high sensitivity ECL solution can be used, such as SuperSignal^TM^ West Femto Maximum Sensitivity Substrate (Thermo Fisher).

### Assay for UFMylation *in vitro*

**Timing: 2–3 days**

A substrate protein can be UFMylated in an *in vitro* system containing UBA5 (E1), UFC1 (E2), UFL1 (E3), DDRGK1 and UFM1 in the presence of ATP. Here, we describe the detailed procedure we used to determine p53 as a novel substrate of UFMylation.

4. Quantify and adjust the concentration of each component (UBA5, UFC1, DDRGK1, UFL1, and UFM1) with Coomassie brilliant blue (CCB).
  ***Note:*** This *in vitro* UFMylation procedure was referred to the protocol established by Kanako Tatsumi (Tatsumi et al., 2010). To reconstitute *in vitro* UFMylation system with all indicated components (including UBA5, UFC1, DDRGK1, UFL1, and UFM1), we quantified the relative concentration of each component using densitometry based on the CCB staining and reconstituted *in vitro* UFMylation reaction by adding all components at equal amount.
  a. Preparation of 12% SDS-PAGE gel.
  b. Load each protein separately about 100–300 ng per well.
  c. Stain gels with staining solution for 2–4 h and de-stain gels with destaining solution for 2–4 h.
  d. Record images and quantify the relative concentrations.
  e. Adjust the loading quantities of each component and make a vitro UFMylation reaction with an equal amount of each component.

5. Perform in *vitro* assay of UFMylation.
  a. Prepare a master mix of *in vitro* UFMylation reaction in PCR tube as follow:

**Table:** 

Reagent	Final concentration	Suggested ratios of DNAs	Amount
ATP (10 mM)	5 mM	N/A	10 μL
UFL1 (100 ng/μL)	5 ng/μL	1	1 μL
DDRGK1 (200 ng/μL)	5 ng/μL	1	0.5 μL
UBA5 (250 ng/μL)	5 ng/μL	1	0.5 μL
UFC1 (1 μg/μL)	5 ng/μL	1	0.2 μL
UFM1 (2.5 μg/μL)	5 ng/μL	1	0.5 μL
MgCl_2_ (100 mM)	10 mM	N/A	2 μL
Substrate protein (700 ng/μL)	17.5 ng/μL	3.5	0.5 μL
PBS-X	N/A	N/A	4.8 μL
**Total**	N/A	N/A	**20 μL**

    ***Note:*** The amount of each component in the table is for one reaction. The amount of substrate protein depends on its concentration based on the CCB staining, and the final volume of each reaction can be scaled proportionally up by the number of reactions in the experiment. We recommend keeping all reagents in an ice bath during the procedure.
  b. Incubate the master mix at 30°C for 90 min.
  c. Add 5 μL of 5× Loading buffer and mix well, then boil for 5 min at 100°C.
  d. Centrifuge at 14,000×*g* for 1 min at 20°C–25°C, then store at −20°C freezer.
  e. Analysis UFMylation of substrate protein by immunoblot, detected by an antibody of the substrate protein as described above.
    ***Note:*** If substrate (p53) protein was modified by UFM1 successfully, at least two bands will be detected (Figure 2).

## Expected outcomes

**Figure 1:**
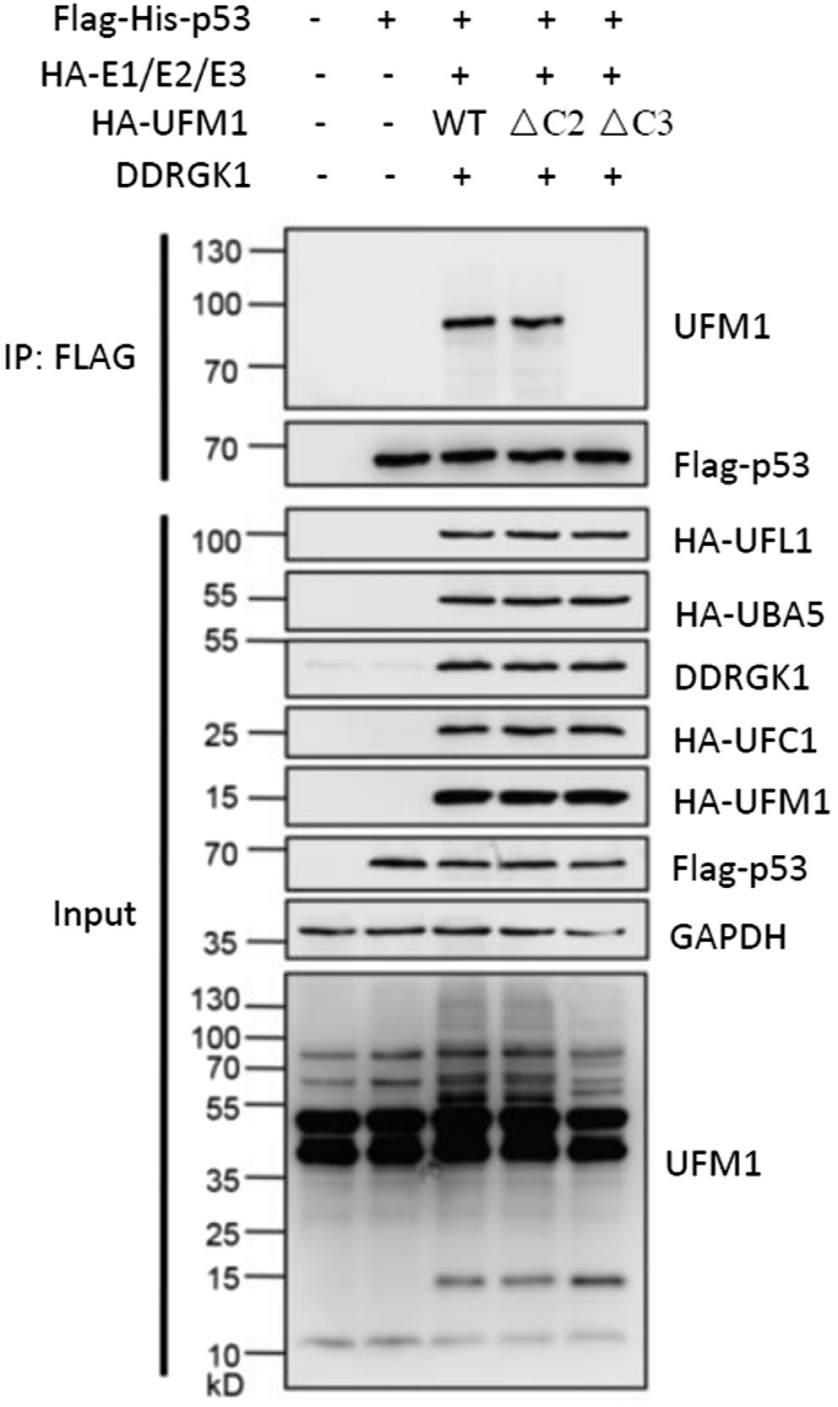
Analysis of p53 UFMylation modification in cells HEK293T cells were transiently transfected by expression plasmids of UFMylation system components and Flag-tagged p53. Cell lysates were immunoprecipitated with anti-Flag M2 Affinity Gel and UFMylation of p53 was analysed by western blot with anti-UFM1 antibody. Data is reproduced from Liu et al. (2020).

**Figure 2:**
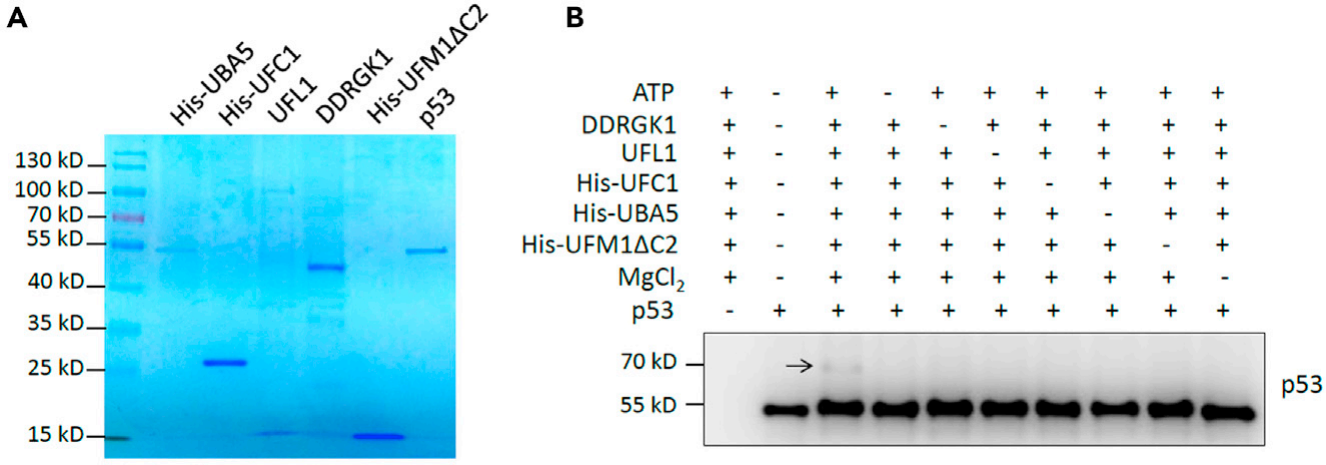
Identification of p53 UFMylation modification *in vitro* (A) Each component were subjected to Coomassie brilliant blue (CCB). (B) In vitro UFMylation of p53. Purified UFMylation components and p53 were incubated in UFMylation buffer. The reaction was terminated by adding SDS sample buffer, and the samples were subjected to western blot with anti-p53 antibody. Data is reproduced from Liu et al. (2020).

The clear mono-UFMylated p53 can be detected by western blot with anti-UFM1 antibody in our UFMylation assay in cells (Figure 1). While in the *in vitro* assay, the weak band of mono-UFMylated p53 and the stronger band of non-ufmylated substrate of p53 can be detected by western blot with p53 antibody (Figure 2).

## Limitations

Owing to the low frequency of modification in cells for some substrates, its UFMylation modification may be hard to detect (Gerakis et al., 2019). Here, we provided an optimized protocol based on transient overexpression of UFMylation components in cells. Although transient overexpression system is widely used in HEK293T cells for detecting various protein modifications including ubiquitin, ubiquitin like (e.g., SUMO, NEDD8, and UFM1), several factors can affect the transfection efficiency, including the cellular state (e.g., cell morphology, cell adherence, cell density, and passage number), quality of PEI solution and plasmids, and the ratio between plasmids and PEI solution, etc. In addition, PEI solution is a cheap transfection reagent with high efficiency HEK293T cells, but it may be not suitable for transfection of other cell types. Alternative transfection approaches including lipofectamine 3000, lentivirus or adenovirus mediated gene delivery may be considered in other cell types. Moreover, UFMylation system is crucial for ER homeostasis, overexpression of UFMylation components may induce ER stress, autophagy, and ER phagy, which may directly or indirectly affect the UFMylation of target proteins. It’s worth mentioning that transient overexpression of UFMylation components may promote the degradation of the target protein in some cases. Lastly, HA-tag and FLAG-tag may alter targeted protein structure, which may interfere with the covalent binding of UFM1.

For these reasons, it is necessary to validate results from the UFMylation assay in cells with the UFMylation assay *in vitro*. Additionally, it’s worth mentioning that mass-spectrum analysis should be used to authorize the modification site within the substrate protein from the immunoprecipitation sediment.

## Troubleshooting

### Problem 1

Absent or weak UFMylated proteins were detected from the UFMylation assay in cells (step 3).

### Potential solution

1) Make sure that the expression plasmids for UFMylation components and substrate were correctly expressed based on the input data from Western blot (Figure 1 lower panel). We recommend agarose gel electrophoresis analysis of all plasmids before each transfection. 2) Include a stronger positive control (e.g., ASC1 (Yoo et al., 2014) or RPL26 (Walczak et al., 2019)) in the UFMylation assay in cells, which can assess whether the experimental procedure was working. In addition, include UFM1-ΔC2 and UFM1-ΔC3 controls to the experimental design (Figure 1 upper panel). 3) Check the whole procedure of immunoblotting, make sure the reagents and operations were correctly used. 4) Use *UFSP2* knockout HEK293T cells, in which the global UFMylation levels are significantly increased due to deficiency of de-UFMylation activity (Walczak et al., 2019). 5) For the weak immunodetection signals, use a high sensitivity ECL solution (e.g., SuperSignal^TM^ West Femto Maximum Sensitivity Substrate, Thermo Fisher).

### Problem 2

The size of UFMylated protein is close to the size of immunoglobulin heavy chains, therefore their signals can be overlapped (step 3).

### Potential solution

Use HRP conjugated secondary antibodies (e.g., HRP Monoclonal Antibody, 3A5C6, Thermo Fisher) that detect only the correctly folded primary antibody, but not denatured heavy chains.

### Problem 3

UFMylated proteins can be reproducibly detected by the assay in cells, but not in the *in vitro* assay (step 5).

### Potential solution

1) Check the quality (expression and concentration) of each component and substrate protein with CCB staining. 2) The degree and efficiency of UFMylation modification for a given substrate may differ to others, the amount of substrate used in UFMylation assay *in vitro* can be optimized. 3) Covalent modification requires a suitable ionic environment and energy, the concentrations of MgCl_2_ and ATP can be optimized. 4) Covalent modification may change the protein structure and disrupt the anti-UFM1 recognition, in this case, it is recommended to use the antibody against substrate and anti-UFM1 antibody in detection of UFMyalted proteins.

### Problem 4

HEK293T cell shows cytotoxic effects after transfection (step 1)

### Potential solution

1) Make sure the plasmids are endotoxin-free. 2) Use fresh HEK293T cells or check cell variability or contaminations. 3) Reduce the amount of PEI (e.g., adjust the ratio of DNA to PEI from 3:1 to 4:1.). 4) Thaw cryopreserved HEK293T cells.

### Problem 5

Unspecific protein band (step 3).

### Potential solution

1) Pre-incubate the cell lysate with Anti-Flag M2 Affinity Gel for 0.5–1 h to remove potential unspecific binding proteins to beads. 2) Rinse PVDF membranes thoroughly before chemiluminescence with ECL solution. 3) Block the PVDF membrane with 5% nonfat dry milk for at least 1 h at 20°C–25°C on a shaker.

## Resource availability

### Lead contact

Further information and requests for resources and reagents should be directed to and will be fulfilled by the lead contact, Yu-sheng Cong (yscong@hznu.edu.cn).

### Materials availability

Materials are available upon reasonable request.

## Data Availability

This study did not generate unique code.
